# Commentary: Vitamin D Deficiency Associated with Cognitive Functioning in Psychotic Disorders

**DOI:** 10.3389/fpubh.2017.00351

**Published:** 2017-12-21

**Authors:** Leo Sher

**Affiliations:** ^1^James J. Peters Veterans’ Administration Medical Center and Icahn School of Medicine at Mount Sinai, New York, NY, United States

**Keywords:** vitamin D, cognition, psychotic, light, testosterone

I read with interest the paper entitled, “Vitamin D deficiency associated with cognitive functioning in psychotic disorders” that was recently published in the *Journal of Clinical Psychiatry* (1). The authors found that vitamin D deficiency affects cognitive function. Vitamin D deficiency was significantly associated with decreased processing speed and decreased fluency.

The observation of the authors of this study is consistent with previous reports suggesting that low serum vitamin D levels may be associated with dementia and other forms of cognitive impairment (2, 3). I would like to expand on this subject.

About 90% of vitamin D is produced in the skin from 7-dehydrocholesterol as a result of sunlight exposure (solar ultraviolet B radiation; 290–315 nm) (4). Most cells in the human body have a vitamin D receptor, and, therefore, vitamin D affects many biological pathways (4).

Inadequate sunlight exposure, often associated with a low dietary intake of vitamin D leads to rickets, a disease involving mostly infants and young children (5, 6). A history of research on rickets is long and interesting. Evidence of vitamin D deficiency in children, such as leg deformities, has been noted in the writings of Soranus of Ephesus and Galen (Claudius Galenus) (both second century CE) (5). In 1645, David Whistler, an English medical student, provided the first scientific description of the signs and symptoms of rickets (5, 6). In the twentieth century, the linking of the knowledge that photosynthesized vitamin D and vitamin D in food were similar was responsible for the defeat of rickets (6).

Studies have shown that light exposure improves cognitive performance and alertness (7, 8). Light can optimize brain function during specific cognitive tasks.

Studies suggest that vitamin D may increase the production of testosterone (9, 10). For example, a significant increase in total testosterone levels, bioactive testosterone, and free testosterone levels was noticed in the vitamin D supplemented men (9). A recent prospective study of a relatively large group of men also showed an increase in blood testosterone levels in individuals who received vitamin D (10). Positive associations between vitamin D and testosterone blood levels were observed by several research groups (11, 12).

Several observations indicate that testosterone supplementation may improve cognitive function (13, 14). For example, one study showed that testosterone replacement therapy led to improvement in attention capacity and psychomotor speed (13). Another study suggests that testosterone replacement therapy improves cognitive performance and mood in men with testosterone deficiency syndrome (14).

These observations suggest that vitamin D and testosterone may partially mediate the cognition-enhancing effect of light in some individuals. Can a combination of light therapy, vitamin D, and testosterone supplementation leads to a significant improvement in cognitive functioning? Further studies of this interesting subject are merited.

## Author Contributions

The author confirms being the sole contributor of this work and approved it for publication.

## Conflict of Interest Statement

The author declares that the research was conducted in the absence of any commercial or financial relationships that could be construed as a potential conflict of interest.
